# Effect of LIBS-Induced Alteration on Subsequent Raman Analysis of Iron Sulfides

**DOI:** 10.1021/acsearthspacechem.2c00051

**Published:** 2022-08-16

**Authors:** Jitse Alsemgeest, Sergey G. Pavlov, Ute Böttger, Iris Weber

**Affiliations:** † Geology and Geochemistry Cluster, Faculty of Science, Vrije Universiteit, de Boelelaan 1085, 1081HV Amsterdam, the Netherlands; ‡ Institute of Optical Sensor Systems, German Aerospace Center (DLR), Rutherfordstr. 2, 12489 Berlin, Germany; § Institut für Planetologie, Westfälische Wilhelms-Universität Universität Münster, Wilhelm-Klemm-Strasse 10, 48149 Münster, Germany

**Keywords:** laser-induced breakdown spectroscopy, Raman spectroscopy, iron sulfides, alteration, plasma interaction, volatiles

## Abstract

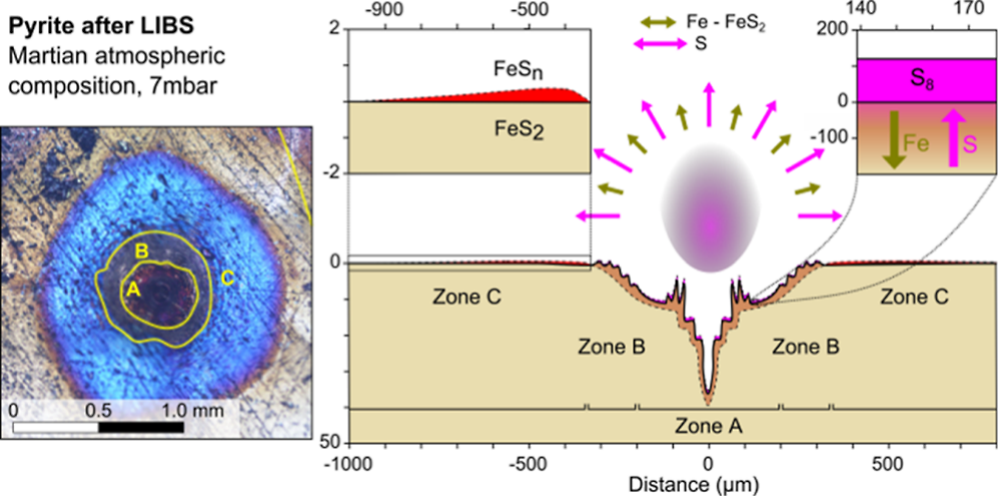

Mineral alteration is a possible side effect of spectroscopic techniques involving laser ablation, such as laser-induced breakdown spectroscopy (LIBS), and is related to the interaction of the generated plasma and ablated material with samples, dust, or ambient atmosphere. Therefore, it is essential to understand these interactions for analytical techniques involving laser ablation, especially for space research. In this combined LIBS–Raman analytical study, pyrite (FeS_2_) and pyrrhotite (Fe_1–*x*_S) samples have been consecutively measured with LIBS and Raman spectroscopy, under three different atmospheric conditions: ∼10^–4^ mbar (atmosphereless body), ∼7 mbar, and Martian atmospheric composition (Martian surface conditions), and 1 bar and Martian atmospheric composition. Furthermore, a dust layer was simulated using ZnO powder in a separate test and applied to pyrite under Martian atmospheric conditions. In all cases, Raman spectra were obscured after the use of LIBS in the area of and around the formed crater. Additional Raman transitions were detected, associated with sulfur (pyrite, 7.0 mbar and 1.0 bar), polysulfides (all conditions), and magnetite (both minerals, 1.0 bar). Magnetite and polysulfides formed a thin film of up to 350–420 and 70–400 nm in the outer part of the LIBS crater, respectively. The ZnO-dust test led to the removal of the dust layer, with a similar alteration to the nondust pyrite test at 7.0 mbar. The tests indicate that recombination with the CO_2_-rich atmosphere is significant at least for pressures from 1.0 bar and that plasma–dust interaction is insignificant. The formation of sulfur and polysulfides indicates fractionation and possible loss of volatile elements caused by the heat of the LIBS laser. This should be taken into account when interpreting combined LIBS–Raman analyses of minerals containing volatile elements on planetary surfaces.

## 1 Introduction

Optical spectroscopy is an essential analytical technique for space research, especially for robotic exploration involving the geology of planetary surfaces.^1−5^ Not only does it allow the identification of normally unreachable targets but also circumvents sample preparation needed for conventional techniques.

Combination of different analytical instruments for investigation of the same sample, as considered, for example, on board the ESA ExoMars rover and in operation on the Perseverance rover (e.g., SuperCam) and Curiosity Rover (e.g., SAM),^6−8^ not only enhances scientific return but also meets additional requirements, such as minimizing and constraining analysis-induced alteration.

In recent years, a particular interest has developed in the combination of two optical techniques: laser-induced breakdown spectroscopy (LIBS, as present on ChemCam and SuperCam of the Mars Curiosity and Perseverance Rovers, respectively) and Raman spectroscopy^6,9−18^ (SuperCam and the ExoMars rover): Both utilize laser excitation and analysis of emitted or scattered light in the visible and near-infrared ranges, allowing for similar technical components that could be compactly integrated. As the first technique provides information on the elemental composition,^19,20^ and the second on crystallinity and molecules,^13^ the combination allows a thorough identification of mineral phases in a single sample. Lastly, the LIBS laser can be used to remove ablated material and clear areas of surface dust,^21−23^ potentially allowing depth profiles through a rock sample.^23−26^

However, as temperatures of the LIBS plasma can exceed 30.000 K^27^ and a shock wave is formed during each measurement,^19,20,28^ the technique might result in alteration of the original material. In several studies on planetary surface alteration,^29−32^ laser ablation was even used as a simulator of micrometeoritic impacts, one of the strongest weathering mechanisms on surfaces of atmosphereless bodies. Furthermore, there can be an interaction between the plasma and particles from the sample, atmosphere, or surrounding dust,^19,20^ which can alter the interpretation of post-LIBS (Raman) spectroscopic data. Therefore, it is essential to understand possible side-effects of LIBS for the interpretation of spectroscopic data and stability of particular materials that are considered for analysis by a LIBS–Raman combined instrument, as well as for other instruments using spectroscopy after LIBS. A few studies have recently considered such potential interaction, focusing on pure metals and oxides, the Gibeon meteorite,^33^ and several geological samples relevant for Mars.^34^ Here, it was found that LIBS-induced alteration occurs,^33,34^ and that heating of the sample is one of the main drivers for this type of alteration.^34^

This work focuses on finding a qualitative mechanism behind any potential alteration and accompanying side-effects of LIBS through subsequent Raman spectroscopic measurements. To do so, LIBS was applied at low power (Section 2.2) to two types of material: pyrite (FeS_2_) and pyrrhotite (Fe_1–*x*_S), under different simulated atmospheric conditions, as well as for pyrite with a layer of ZnO simulating dust material. These minerals contain sulfur, a volatile element, and iron, whose oxides are detectable by Raman spectroscopy. Furthermore, the two elements are common on Mars^22,35−38^ and other solar system bodies such as asteroids, and are found as dark phases in meteorites (e.g., as in Schrader et al.^39^). Their relatively simple chemistry allows observation of potential interaction with a Martian-like (CO_2_) atmosphere and dust particles.^40−42^ Raman spectroscopy was performed on the samples before and after applying LIBS to track changes caused by the LIBS plasma. Raman data were combined with optical microscopy to determine the alteration areas and mechanisms, and to show how minerals in a Martian-like environment and atmosphereless bodies are likely to suffer changes from LIBS in the combined LIBS–Raman methodology.

## 2 Methods

Natural samples of pyrite (FeS_2_) and pyrrhotite (Fe_1–*x*_S) were prepared using a diamond saw and SiC (silicon–carbide) sandpaper for polishing (grits of 80–2400). The samples were then investigated by subsequent micro-Raman spectroscopy, LIBS, and again micro-Raman spectroscopy to track, in detail, potential changes caused by the LIBS measurement. From previous Raman measurements of the unaltered sample surface, it was noted that several minor mineral impurities exist within the two sample types (pyrite, calcite, anhydrite, and rutile, as well as traces of quartz, anatase, barite, pyrrhotite, pentlandite, chalcopyrite, magnetite, and further minerals without identifiable Raman spectrum). Therefore, only the most homogenous (pure pyrite and pure pyrrhotite) sections were used for the LIBS measurements. Possible effects of impurities are discussed in Section 4.4.

All measurements were done under fixed atmospheric conditions, using a single sealed sample chamber suitable for both Raman spectroscopy and LIBS, from which the sample was not removed during any of the measurements. Three atmospheric conditions were used: 2 × 10^–4^ mbar (vacuum), 7.0 mbar of Martian-like atmospheric composition (premixed 95.6% carbon dioxide, 2.7% nitrogen, 1.6% argon, 0.15% oxygen), and 1.0 bar of Martian-like atmospheric composition. These conditions were used to avoid interaction with the terrestrial atmosphere and to represent asteroid, lunar, or other atmosphereless surface conditions (vacuum); to check for interaction under Martian conditions (7.0 mbar); and to check if interaction with the atmosphere plays a role at higher pressures (1.0 bar). Additional experiments were done for pyrite covered with a 300 ± 60 μm layer of zinc oxide (ZnO) to investigate the potential interaction between LIBS plasma and dust, under 7.0 mbar at Martian atmospheric composition. ZnO was chosen to represent dust, as it has low reactivity, and Zn has low ionization energy and is not present as impurities in the samples. For this reason, the occurrence of Zn-lines in the emission spectrum of the plasma can be used for the characterization of the dust ablation and its plasma. Each measurement was repeated to check for consistency. No safety hazards, which are not normal or expected for experiments involving LIBS or Raman-spectroscopy, were encountered.

### 2.1 Raman Spectroscopy

Raman spectroscopy was performed with a WITec alpha 300 confocal Raman microscope and a frequency-doubled Nd/YAG laser at 532 nm. Measurements were accumulated over multiples of 10 s, using a laser power of 1.0–1.2 mW and an objective of 10x/0.25 (laser spot diameter <1.5 μm). Reflected and Raman-scattered was collected through the same objective (distance to sample ∼12 mm), and collected using a WITec Ultra High Throughput Raman Spectrometer 300 with a 600 mm^–1^ grating providing a spectral resolution of about 10 cm^–1^ (4 cm^–1^/pixel). The spectrometer CCD detector was cooled to −60 °C. Further analysis was done with the software WITec Project Four. Raw data have been processed as detailed in the Supporting Information—Data Processing.

### 2.2 Laser-Induced Breakdown Spectroscopy

LIBS was performed using a Q-switched multimode (quasi-flat-top) Nd-YAG laser at 1064 nm and a simulation chamber. The laser output has a maximum output energy of 240 mJ (6 ns pulses) that was reduced by an attenuator (a set of neutral density filters) to ∼4 mJ on the sample surface to obtain low ablation and excitation rates, namely a factor about 2 above the threshold for observation of plasma emission at the lowest pressure (vacuum). The irradiated area on the samples was about 0.07 mm^2^. The laser beam heated the samples at an almost perpendicular direction (∼5° incident angle). Postablation metrics, such as ablated volume, deposited material, and plasma temperature (see Section 4.3 and Supporting Information for details) were taken as merits for the induced ablation and plasma excitation. Each measurement consisted of 50 shots with a repetition rate of 10 Hz. Atomic emission spectra were then collected at a wavelength range of 281–900 nm (resolution of 21–52 pm and resolving power of 14,500) using an Aryelle Butterfly echelle spectrometer with an Andor iStar ICCD detector. The acquisition of the plasma emission has been optimized empirically to obtain the best signal-to-noise of Fe emission lines for vacuum conditions, resulting in a laser pulse delay of 100 ns and a gate time of 5 μs, similar to those used in Pavlov et al.^43^ Raw data have been processed as detailed in the Supporting Information—Data Processing. Elemental compositions and plasma temperatures were derived using the NIST Atomic Spectra Database.^44^

## 3 Results

### 3.1 Elements Detected by LIBS

**Figure 1:**
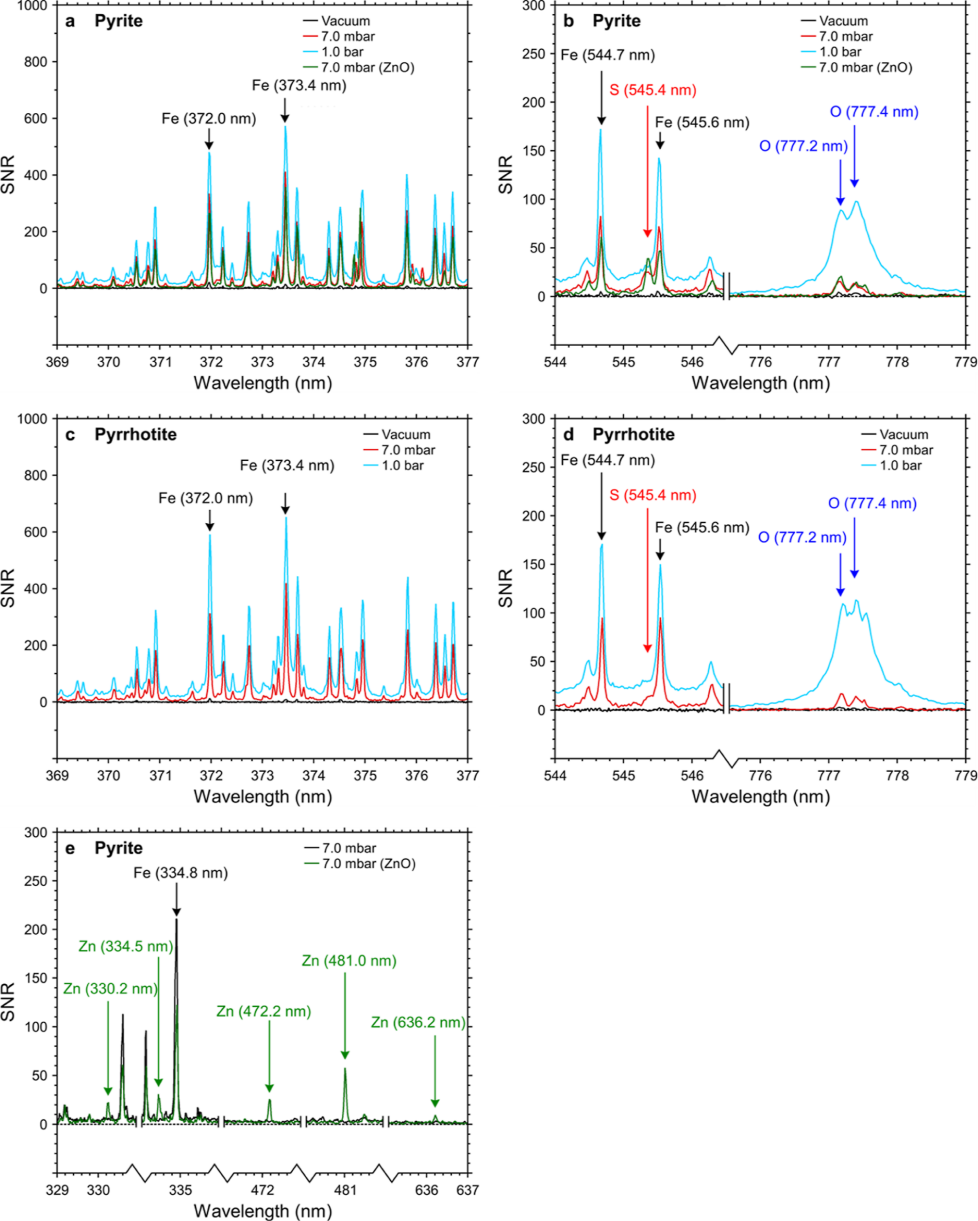
Selected parts of LIBS spectra measured on pyrite (a,b,e), pyrrhotite (c,d), and pyrite with a ZnO dust layer (e), under Martian atmospheric composition and different pressures. Signal-to-noise ratios and background subtraction as detailed in the Supporting Information—Data Processing. Annotations show interpretations of elemental lines. SNRs for Fe are weak under a vacuum but do not differ much between 7.0 mbar and 1.0 bar. The oxygen SNR in both pyrite (b) and pyrrhotite (d) is significantly stronger under 1.0 bar than under 7.0 mbar, indicating the interaction of the LIBS plasma with CO_2_ in the atmosphere.

The LIBS spectra for both pyrite and pyrrhotite (Figure 1) consist dominantly of atomic Fe-lines (Figure 1a,c); neutral Fe was detected at all pressures, whereas singly ionized Fe was only detected at medium and higher ambient pressure (7.0 mbar to 1 bar). A single, weak line was detected at 545.4 nm (Figure 1b,d) which could represent an S (II) line,^44^ but no other lines were detected (several S lines are overlapping with stronger Fe lines) and no further conclusions can be drawn on sulfur in the plasma. Traces of additional elements were detected in the form of weak or few lines (for pyrite: H, O, Mg, K, Na, Si, and Ti; for pyrrhotite: H, O, Ni, and Si) at 7.0 mbar and 1.0 bar in Martian-like atmospheric composition, although these were not observed under vacuum conditions. Notably, the intensity of the oxygen lines increases significantly between 7.0 mbar and 1.0 bar for both pyrite and pyrrhotite (Figure 1b,d).

For the experiment with the ZnO dust layer, the spectra contain several neutral atomic Zn-lines (Figure 1e), indicating the population of Zn excited states in the plasma. Considering probabilities of intracenter transitions for Zn and Fe under similar measurement conditions^44^ (e.g., above 108 s^–1^, see the Supporting Information), the reduced signal is more likely linked to a different number of shots over which plasma emission is accumulated because dust may be removed after the first few shots^21^ (further effects discussed in Section 4.3). Additionally, a slight increase in emission intensity (1.5–4x) was noted in the O-triplet at 777 nm (Figure 1b). Reduced intensity of Fe-related transitions with respect to the measurement at Martian atmospheric conditions on pyrite may also be related to the ZnO dust obscuring the sample during the first few pulses.^21^

### 3.2 Alteration Zones

**Figure 2:**
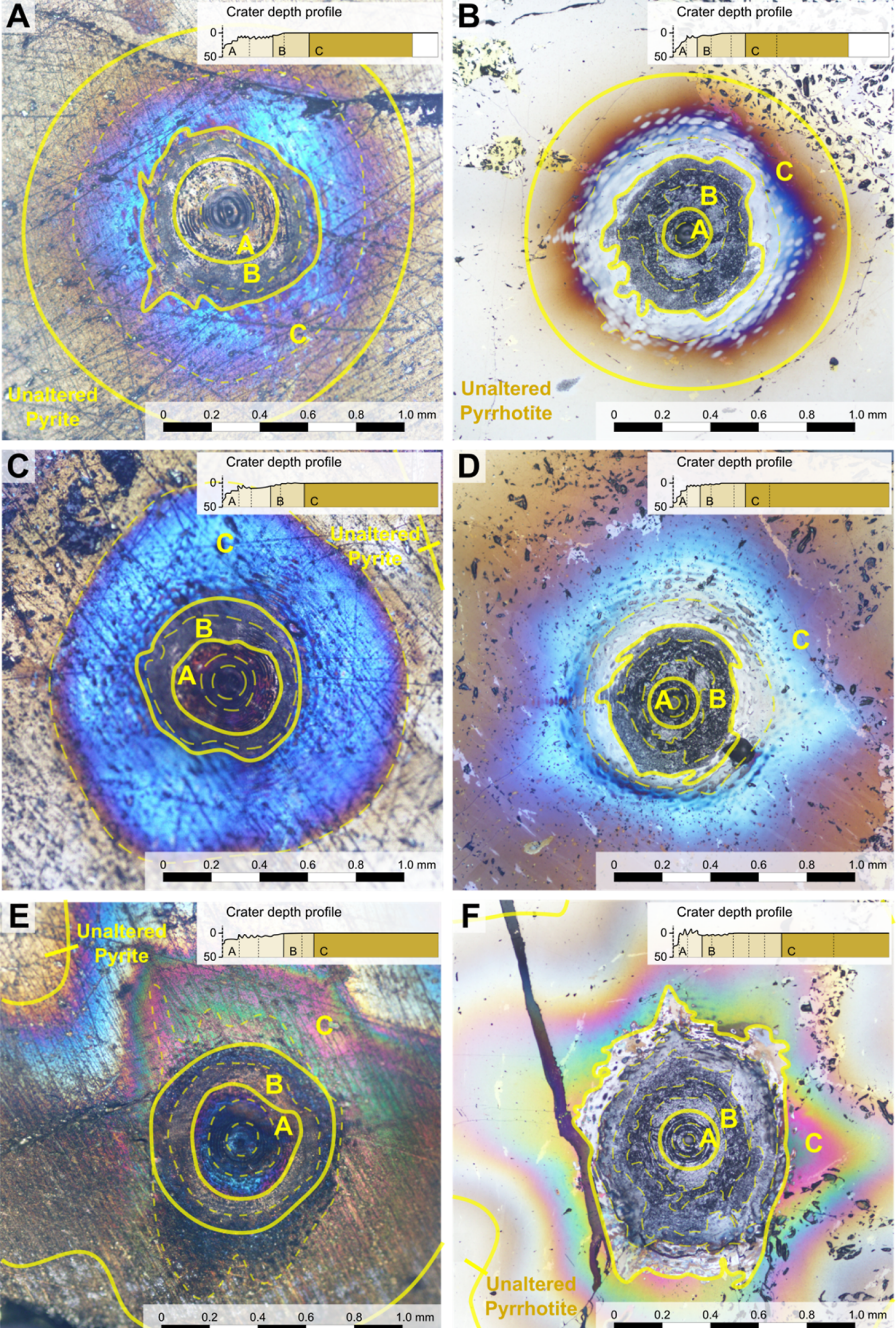
Overview of alteration caused by LIBS in different materials, under different conditions. Microphotographs: A: Pyrite, vacuum; B: Pyrrhotite, vacuum; C: Pyrite, 7 mbar, Martian atmospheric composition; D: Pyrrhotite, 7 mbar, Martian atmospheric composition; E: Pyrite, 1.0 bar, Martian atmospheric composition; and F: Pyrrhotite, 1.0 bar, Martian atmospheric composition. Craters are divided into zones, labeled A–C, based on their morphology. In all circumstances, a thin film is created that causes distinct color changes, which consists of polysulfides (all conditions) and magnetite (1.0 bar). On top of this, sulfur is produced in pyrite (zone A), causing the color to change to blueish purple.

Throughout all experiments, the LIBS plasma caused a similar type of alteration of the sample surface (see Figure 2 for details). This alteration was not constant over the entire area of the crater, and therefore, the different effects are described for different zones in the crater. Three semicircular zone types were identified, based on visual characteristics: color and brightness. The first zone (zone A) occurs in the inner part of the crater structure and is characterized by a rough structure without preferred orientation. For pyrite (Figure 2a,c,e), an additional color change was noted from red to purple, which is more pronounced in the innermost parts of the crater. It was noted that this color change diminished over the day, and was not present for pyrrhotite (Figure 2b,d,f). The second zone (zone B) shows concentrical ripples and incorporates the edge of the crater. The third zone (zone C) lies outside of the crater edge and shows distinct color changes corresponding to thin-film interference, under which the original material is still visible. Notably, the third zone was different in the pyrrhotite measurement at 1.0 bar, where it shows a white, reflective layer as well as spots.

**Figure 3:**
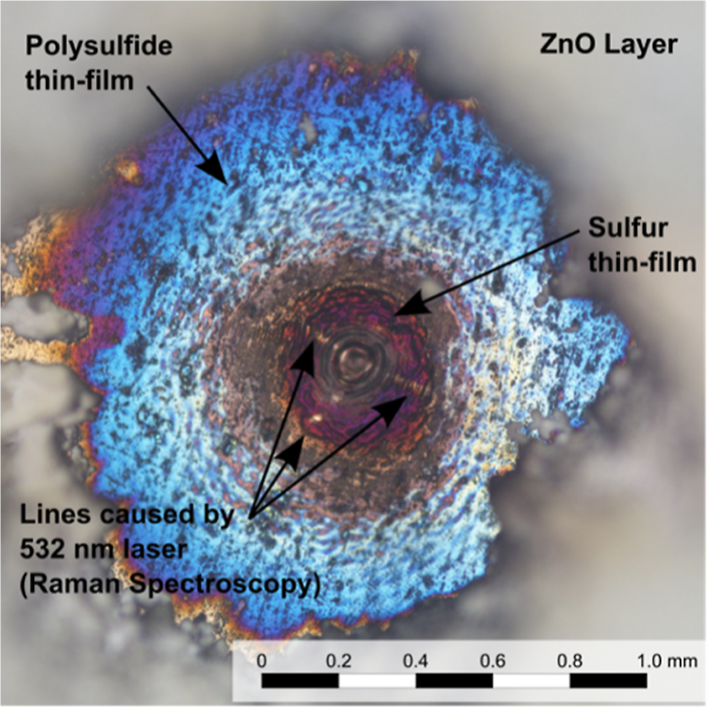
Representative photograph of the resulting crater from the experiment with LIBS on pyrite covered with a ZnO dust layer at 7.0 mbar Martian atmospheric composition. Dust is successfully removed, but the developed thin film has a similar size to the removed portion of dust. Alteration is the same as for pyrite, 7.0 mbar, without ZnO (Figure 2b).

The dust experiment (Figure 3) yielded similar results to the experiments on pyrite at 7.0 mbar. Remarkably, the extent to which dust is blown away is limited to the extent of alteration zone C.

### 3.3 Changes in the Raman Spectrum

**Figure 4:**
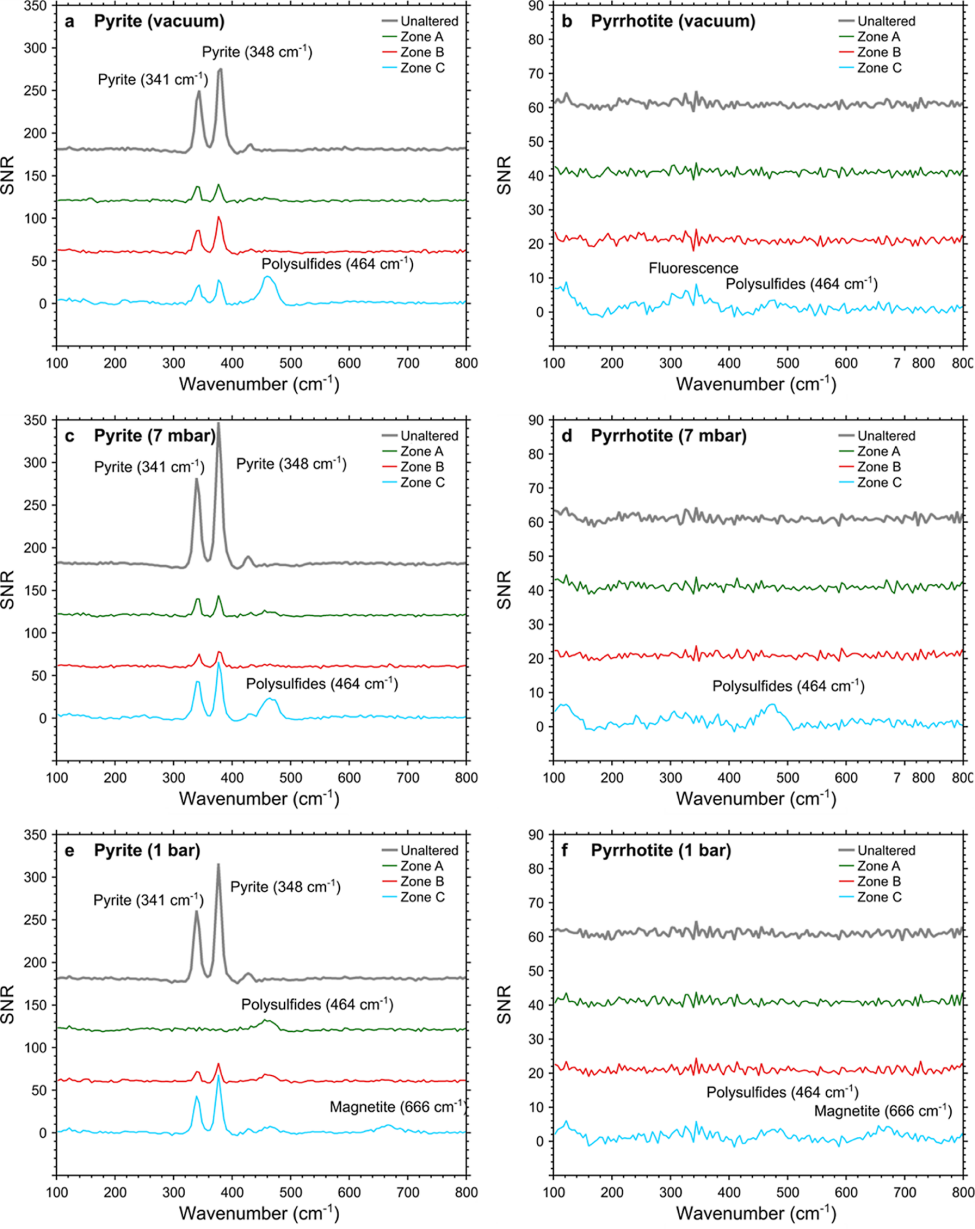
Selected Raman spectra representing different alteration zones in pyrite (A,C,E) and pyrrhotite (B,D,F) caused by interaction with the LIBS plasma. Measurements of unaltered pyrite and pyrrhotite are shown in grey. All measurements represent averages of time series (10 × 10 s). For spectra of sulfur, only measured in the first spectrum of the 10 × 10 s series, see Figure 5. Each spectrum is shifted at +60 SNR (pyrite) or +20 SNR (pyrrhotite), SNR and background subtraction as detailed in the Supporting Information—Data Processing. The artifact at ∼120 cm^–1^ is related to the imperfect filtering of Rayleigh-scattered light from the Raman laser.

**Figure 5:**
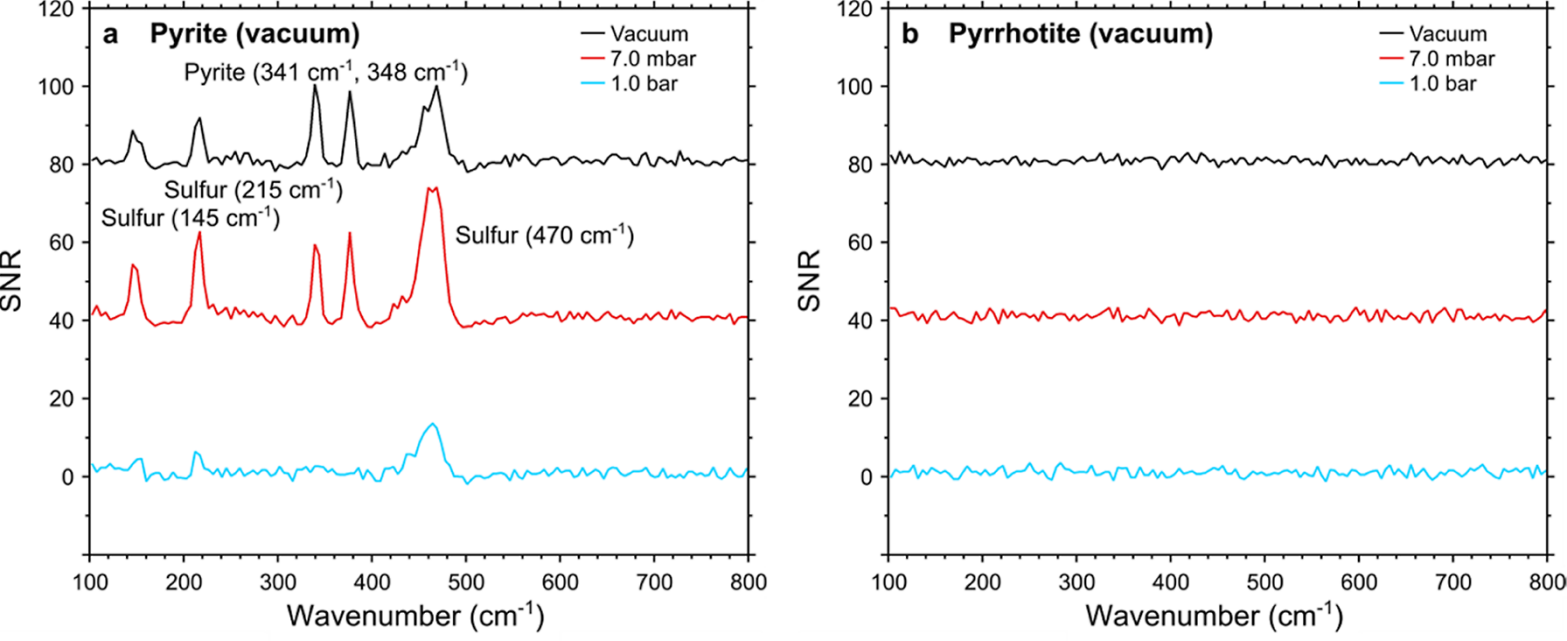
Representative Raman spectra of alteration zone A of pyrite (a) and pyrrhotite (b). Accumulation of the first 10s of the Raman measurement, still showing the sulfur spectrum that is not visible in the time series displayed in Figure 3. SNR and background subtraction as detailed in the Supporting Information—Data Processing. Note that sulfur is only produced for the LIBS measurements on pyrite, whereas pyrrhotite shows no clear Raman features whatsoever.

For pyrite (Figure 4a,c,e), original (before LIBS) Raman spectral features at 341 and 348 cm^–1^ became less pronounced toward the inner crater zones as a result of the LIBS measurement. Furthermore, within zone A and zone C, several new peaks were identified, related to the color changes noted in Section 3.2. As previously mentioned, the least alteration was found under vacuum conditions, whereas most alteration was found under 1.0 bar. Within zone A, Raman peaks were found at 145, 215, and 470 cm^–1^, although these disappeared after a few seconds of laser irradiation, even at low laser powers (<1.0 mW, Figure 5). Together with the diminishing color change, this is interpreted as elemental sulfur.^45,46^ Within zone C, Raman peaks were found at 460 and 660 cm^–1^, corresponding to polysulfides^47−49^ and magnetite,^45^ respectively. Notably, magnetite was only produced under 1.0 bar (Martian atmospheric composition), whereas polysulfides and sulfur were produced under all atmospheric conditions.

For pyrrhotite (Figure 4b,d,f), signal-to-noise ratios (SNR) of deposited material are significantly lower than for pyrite (Figure 4a,c,e). However, the characteristic band of polysulfide, centered at 464 cm^–1^ is present in zone C throughout all conditions but is best distinguished at 7 mbar. At 1 bar, the broad band for magnetite at 666 cm^–1^ can also be distinguished. In none of the scenarios, elemental sulfur was deposited on the original pyrrhotite surface, and fewer polysulfides were formed than with the pyrite experiments (Figure 5).

The dust experiment yielded the same alteration types as for pyrite at 7.0 mbar.

## 4 Discussion

### 4.1 Drivers for Alteration

**Figure 6:**
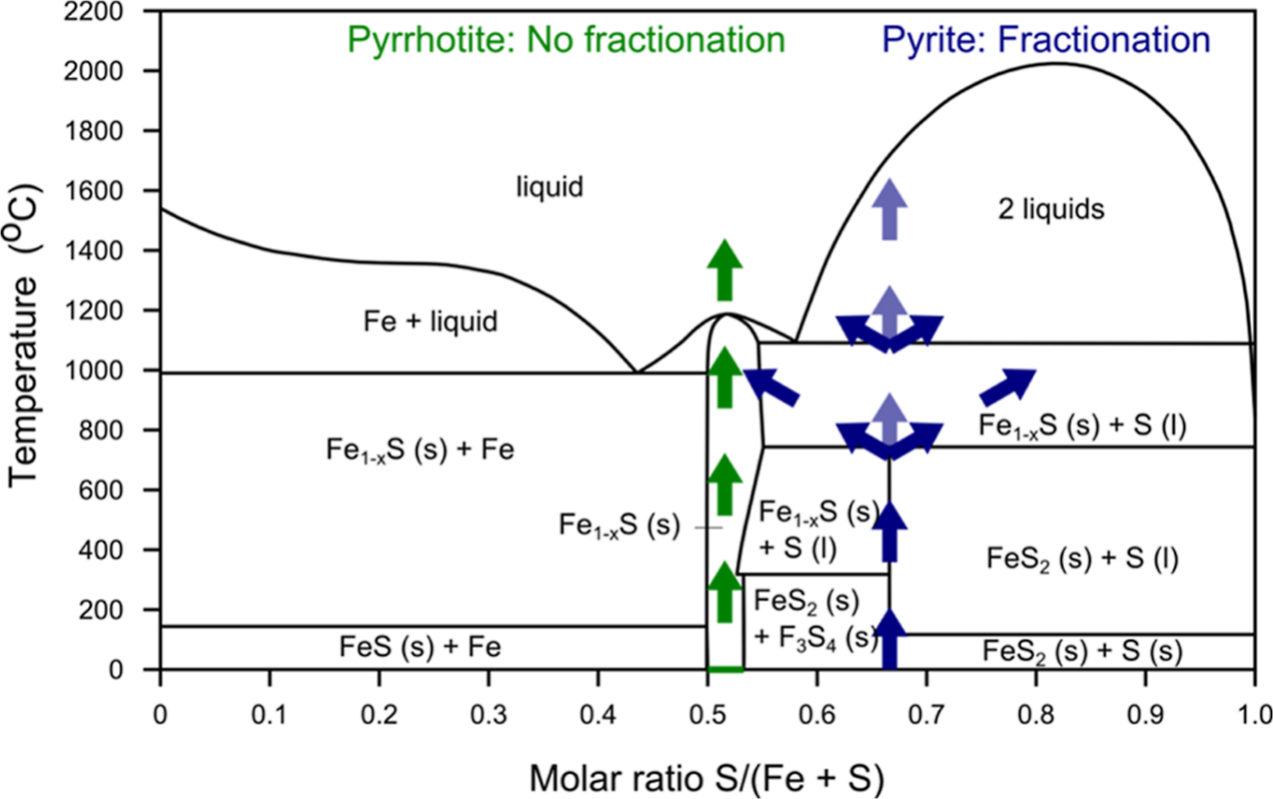
Simplified phase diagram of iron sulfides (*P* = 1 bar), showing heating of pyrite (blue) and pyrrhotite (green). Adapted from published data.^50,51^ Upon heating of pyrrhotite, no (or few, depending on composition) phase changes are encountered until about 1100–1200 °C, whereas pyrite first decomposes into pyrrhotite and liquid sulfur, and then into 2 immiscible liquids. As sulfur has a lower density than any iron-rich components, any melt layer created by the heat of the LIBS layer likely fractionates. This leads to the enrichment of sulfur toward the upper part of the layer (Figure 7a), explaining the detection of sulfur in the Raman spectrum of the inner parts of the crater only.

The experiments on pyrite and pyrrhotite both produced polysulfides and magnetite, whereas sulfur was only produced for pyrite. Pyrrhotite, instead, developed white spots which did not give a distinct Raman spectrum. The production of sulfur for pyrite only may indicate that heat is the main driver for alteration. Following a standard state phase diagram of iron sulfides (Figure 6),^50,51^ heating of pyrite should lead to the following reaction at about 750 °C
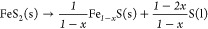
1

This means that for pyrite, the formation of a sulfur-containing (partial) melt layer is possible at the heated sample surface, where a surplus of sulfur indicates that some fractionation even took place within this melt layer. However, sulfur is not completely stable at low pressures,^52^ as also noted through the diminishing color over the day, and therefore, it likely partially evaporated during the experiment as well.

The different reaction path for pyrrhotite explains the lack of elemental sulfur for this mineral. However, it does not explain the polysulfides, produced for both minerals.

**Figure 7:**
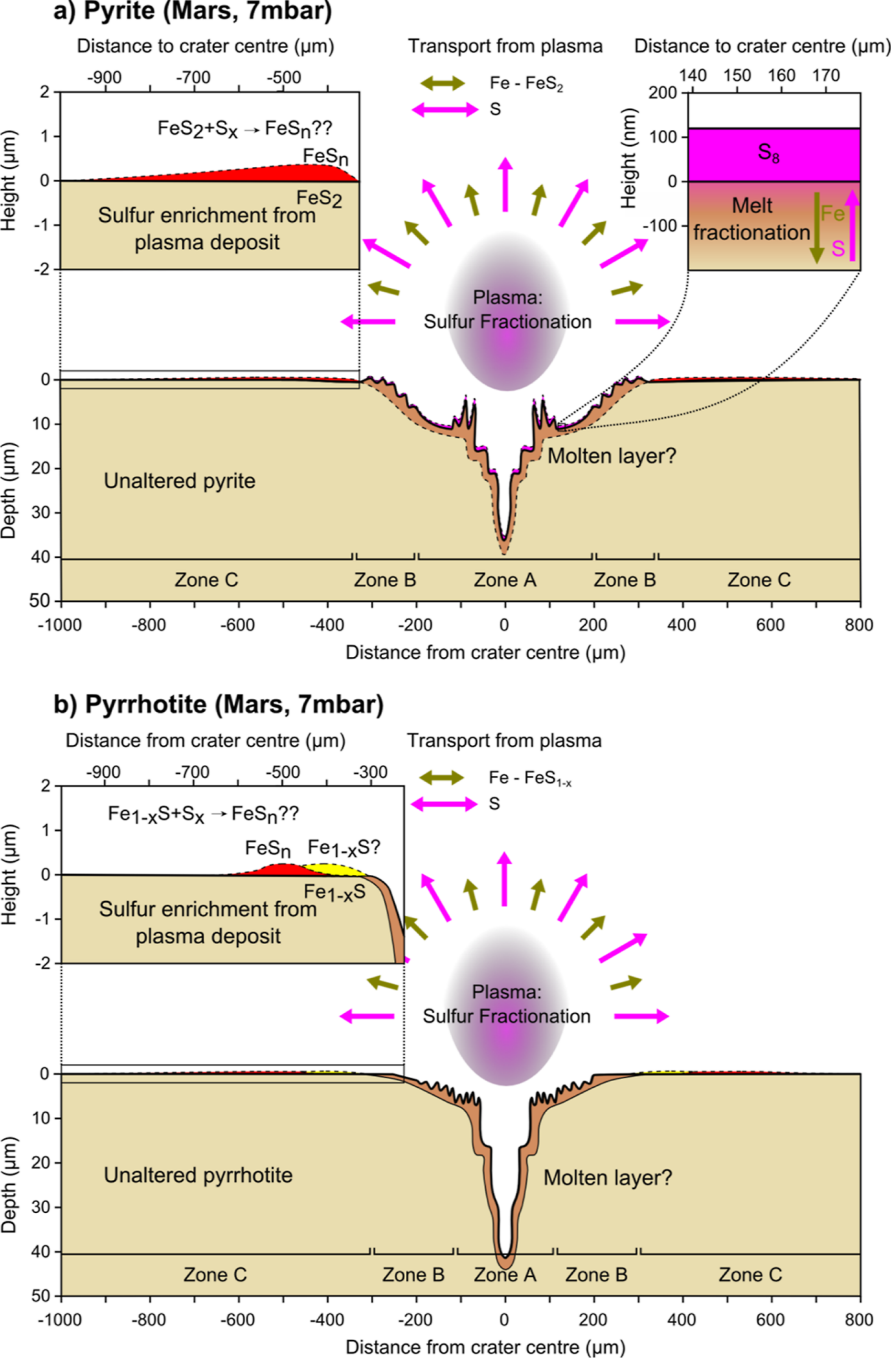
Conceptual model of alteration of pyrite (a) and pyrrhotite (b) at 7 mbar, showing the behavior of the main components. A: for pyrite, melt fractionation in Zone A (Figure 2c) explains the occurrence of elemental sulfur (see also Figure 6). However, this does not explain the occurrence of a thin film of polysulfides in zone C, which can only be explained by the enrichment of sulfur as a result of fractionation of the plasma. The still hot sulfur then likely reacts with either iron in the plasma, or with pyrite upon deposition to form the polysulfide thin film. B: for pyrrhotite, no melt fractionation occurs (Figure 6), and similarly to pyrite, sulfur fractionates out of the plasma and then reacts with either a small amount of iron in the plasma or with pyrrhotite to form a polysulfide thin film in zone C.

Polysulfides may be explained by elements present in the LIBS plasma. For both pyrite and pyrrhotite, the main expected elements in the plasma are sulfur (S) and iron (Fe). First, the plasma may be enriched in sulfur due to sulfur evaporation at the sample surface. Second, S particles are lighter than Fe particles and will, therefore, be transported further away than Fe particles with the same kinetic energy. This may lead to the fractionation of S from Fe away from the crater center (Figure 7), regardless of plasma-related parameters. This can in turn lead to a surplus of hot sulfur in the outer parts of the crater, which may either have formed iron polysulfides through recombination with remaining Fe-particles in the plasma or possibly through the reaction of sulfur particles with original pyrite and pyrrhotite (Figure 7).

Magnetite was only found at 1.0 bar CO_2_, which, together with the intensity of the oxygen signal (Figure 1b,d), indicates recombination of Fe-particles with O-particles from the atmosphere. Similarly, recombination of S and O is expected at 1.0 bar, which would lead to the removal of sulfur in the form of a gas phase (SO_2_)—however, the presence of the polysulfide layer indicates that this recombination is not sufficient to eliminate large amounts of sulfur. Lower amounts of atmospheric oxygen at 7.0 mbar make this effect negligible for Martian-like conditions; although, it should be taken into account for the combined LIBS–Raman application on planetary surfaces with higher atmospheric pressures. No recombination occurred with respect to the ZnO dust layer in Martian-like atmospheric conditions, despite the clear removal of the layer.

In summary, the two drivers that play a role in LIBS-induced alteration are heat (>750 °C in a melt layer) and atmospheric recombination. However, the latter only plays a minor role when considering Martian-like conditions or atmosphereless bodies.

### 4.2 Ablation and Deposition

Results indicate a significant alteration in the Raman spectrum (Figures 4 and 5). For a sense of how much of the ablated material is redeposited and how much is lost, an estimate is needed of the crater volume and the thickness of the alteration layer.

The crater volume can be estimated by integration over the crater profiles. This indicates ablated volumes of roughly 0.5–1.7 mm^3^ (Table 1), averaging about 0.01–0.03 mm^3^/shot. With a crater width up to 0.8 mm, this gives an average ablation of 0.02–0.06 mm/shot. This compares well to other studies,^53−55^ where the average rate was between ∼0.005 mm/shot (100 shots with ChemCam instrument on basaltic targets, Vickers Hardness ∼1000) and ∼0.07 mm/shot (50 shots with ChemCam replica laser at 6.7 mbar and 3 m distance in Martian soil simulant, Vickers Hardness ∼17–46).

**Table 1:** Estimated Ablated Volumes^a^ (mm^3^) for Both Minerals

atmospheric condition	pyrite	pyrrhotite
vacuum	1.51	0.89
martian atmosphere, 7 mbar	1.74	0.68
martian atmosphere, 1 bar	1.26	0.53

a Based on the integration of the found crater depth profiles around the central axis of the crater.

The thickness of the alteration layer can be estimated from optically determined interference colors. All experiments resulted in an alteration zone (C) displaying thin film interference. This is caused by reflection at the top and bottom of a thin film with a different refractive index than the underlying material. This leads to a path difference, depending on the thickness of the thin film (traveled path of light), refractive index, and the wavelength of the light. Different wavelengths provide either constructive or destructive interference, leading to a specific color depending on the thickness and type of material of the thin film. However, color may also arise from absorption, dependent on the thickness and transparency of the thin film, and the two effects are taken into account in the interpretation of the thin film thickness below.

As mentioned in Section 3.3, the thin films consist of polysulfides (vacuum and Martian atmospheric conditions) and magnetite (1.0 bar, Martian atmospheric composition). Interference colors were in the inner zones of craters in pyrite (7 mbar and 1.0 bar) and were related to an elemental sulfur thin film.

For the polysulfides, the optically determined maximum interference color is second-order blue (Figure 2), indicating a maximum path difference Γ of 600–700 nm.^56^ Assuming a refractive index *n* between 1.5 and 2.5 (exceeding indices for pyrite, 1.73, and sulfur, 2.08), and using Γ = 2*nd*,^56^ a maximum layer thickness *d* can be calculated to be 90–195 nm. Transparency of the polysulfide thin film for different wavelengths is unknown, but because the traveled path is <400 nm, absorption is likely minimal and the color is mainly related to thin-film interference.

Similarly, the path difference for the magnetite layer is roughly 1800–2000 nm (fourth-order green). With a refractive index of about 2.42,^57^ this indicates a maximum *d* of 350–420 nm. However, as magnetite is opaque, the preferential transmission of blueish purple colors should be taken into account already for path differences >100 nm.^58^ This could explain that the fourth-order green color is darker in appearance than would be expected,^56^ although the distinct interference color pattern still allows accurate estimation of the path difference and, therefore, the layer difference.

For the elemental sulfur thin film, maximum path differences were found to be roughly 500 nm (7.0 mbar, purple) and 600–700 nm (1.0 bar, purple). With a refractive index of about 2.08,^59^ this indicates sulfur layer thicknesses of roughly 120 and 144–168 nm, respectively. Furthermore, as sulfur is transparent at thicknesses <30 μm,^60^ the color can be attributed to thin-film interference alone.

The thin-film area is highly variable in size and shape, and it is not clear whether polysulfides form through recombination of Fe and S or reactions between pyrite or pyrrhotite with hot sulfur. Therefore, it is not possible to provide a direct estimate of the redeposited part of the ablated material. However, to provide a rough sense, even if a maximum thickness (∼420 nm) is assumed over a large area (4 mm^2^), the resulting volume will be in the order of 0.006 mm^3^, 2 orders of magnitude lower than the smallest crater volume. Thus, it is safe to assume that most of the ablated material did not get redeposited as a thin film and may have been dispersed, possibly partly as a gas phase, throughout the sample chamber.

### 4.3 Plasma Temperatures

Plasma temperatures were calculated using the two-line method and the Boltzmann plots^19^ based on the different elemental lines found in the LIBS spectra of pyrite and pyrrhotite (see Supporting Information for details). Temperatures derived from Fe-lines, present throughout all spectra, are collected in Table 2. For pyrite, the plasma temperature is the lowest for vacuum (∼5200 K), the highest for 7 mbar (∼7150 K), and intermediate for 1 bar conditions (∼6950 K). Similar results were found for pyrrhotite (∼5200 K for vacuum, ∼7000 K for 7 mbar, and ∼6550 K for 1 bar conditions). Significantly higher temperatures were derived in the analysis of the spectra in the laboratory Martian simulation conditions (12,000–17,000 K^53^) and in situ measurements (12,000–35,000 K^61^) with the ChemCam instrument, which utilizes higher energy Gaussian-like beams. We note here that plasma temperature cannot serve as a proper metric for an accurate comparison between observations at different pressures due to its dynamic change on a μs time scale.^62^ The chosen acquisition parameters were aimed for accurate comparison of emitted intensities rather than for comparison of plasma temperatures at different ambient pressures. The 5 μs acquisition window was chosen to integrate over the entire lifetime of the plasma, whereas specifically chosen shorter acquisition windows would be desired for accurate comparison of peak plasma temperatures. The 100 ns delay was necessary to not saturate the ICCD detector with the continuum bremsstrahlung emission at high pressure, but may lead to the lower values of derived temperatures for vacuum and related short-lived plasmas (∼100 ns).^63^

**Table 2:** Estimated Temperatures Based on Fe-Lines in LIBS Spectra^a^

conditions	*T* (K)^b^ (two lines)	*T* (K)^b^ (Boltzmann plot)
Pyrite		
vacuum	5170 ± 250	5200 ± 480
7 mbar	6600 ± 340	7150 ± 770
1 bar	6260 ± 260	6950 ± 700
Pyrrhotite		
vacuum	5210 ± 360	5190 ± 590
7 mbar	6360 ± 370	7000 ± 750
1 bar	5730 ± 310	6550 ± 600
		

a See Supporting Information for details on plasma-temperature calculation.

b Temperatures represent mean values calculated over two spectra.

For the ZnO-dust experiment, additional estimates were done for Ti (7300 ± 1100 K) and Zn (∼6500 K). While the latter estimates suffer from a limited number of observed transitions, the temperature estimate for Ti is the same, within error bars, as those derived for Fe lines. Significantly lower estimates for temperature through atomic Zn transitions may be partly related to Zn being present in the form of a powder: the powder is easily blasted away in the first few (of 50) pulses,^21^ and therefore, Zn may be concentrated close to the sample surface and at ∼5–10 mm from the center of the plasma plume,^21^ where electron densities are lower, collisional processes are less frequent, and plasma temperatures are lower^64^ than in the plume center.

### 4.4 Other Origin of Oxygen?

An increase in the oxygen LIBS signal, and formation of magnetite, was detected at higher pressures (Figure 1b,d), which is interpreted to be related to the breakdown of CO_2_ from the atmosphere. To verify this, the following explanations need to be excluded; (1) oxygen from impurities in the samples, (2) oxygen from ZnO powder in the ZnO dust experiment, and (3) pressure-dependence of oxygen signals.

First, although only homogenous (“clean”) sections were used (Section 2), the samples contain impurities. These make up ∼ 10% of the pyrite sample and ∼45% of the pyrrhotite and, respectively, consist of (in order of importance) calcite, anhydrite, rutile, and other oxides and pentlandite, chalcopyrite, and magnetite. Assuming these impurities are the main source of oxygen, they are expected (1) not to be distributed uniformly in pyrite and pyrrhotite and (2) to behave similarly under 7.0 mbar and 1.0 bar, with SNR relative to the impurity density. The O-triplet at 777 nm is clearly present at 1.0 bar and is significantly stronger than at 7.0 mbar, and is similar in pyrite and pyrrhotite. This indicates that, at least at 1.0 bar, there is a significant source of oxygen that is unrelated to impurities.

Second, for the ZnO experiment, oxygen may also be derived from the ZnO dust, which may explain the detectable increase in the oxygen signal (Figure 1b). However, this increase could alternatively be related to a LIBS signal enhancement effect related to the particle size of the ZnO dust,^65,66^ causing increased plasma effectivity. Furthermore, there is a limited reproducibility in the thickness of the ZnO layer, and therefore, the relative intensities of Fe, Zn, and O vary more strongly in experiments with the layer (O/Fe ratios vary ∼27%) when compared with experiments without the ZnO layer (O/Fe ratios vary ∼3%).

Third, oxygen atoms have relatively large ionization energy, are therefore generally less excited than iron, and are expected to exhibit lower emission signals.^67^ Hence, under similar conditions, for similar transition probability of intracenter transitions, the intensities of oxygen lines are generally lower (up to factor five) than those of iron lines.^44^ The large ionization energy means that low collision rates in a vacuum may not be sufficient to obtain an oxygen signal. However, at 7 mbar and 1.0 bar, pressures are sufficient, and the O-signal (Figure 1b,d) should scale with the Fe-signal (Figure 1a,c). However, the strong enhancement of oxygen SNR in the LIBS spectra at 1.0 bar indicates the influence of the CO_2_-rich environment on the postablation chemistry, especially considering its lower relative intensity compared to Fe-lines throughout the spectrum.^44^

The origin of oxygen could be further verified by comparing signals for C and S to relate the increase of SNR either directly to CO_2_ or increased effectivity by comparison with an element of comparable electron affinity. However, this is not possible as the strongest lines for C and S are outside of the spectral range (in the ultraviolet), explaining why little to no C or S could be detected in these experiments.

### 4.5 Implications for Space Research

#### 4.5.1 Current and Future Instrument Interpretation

LIBS has been applied at levels just above the laser ablation threshold, and micro-Raman has been applied to investigate macroscopic alteration in detail. This was done to (a) investigate the minimum alteration related to LIBS operation and (b) differentiate between different sections around the LIBS crater. Instruments currently on Mars, however, use different parameters for both LIBS and Raman operation, such as different laser energy distribution, a factor of 2–3 higher LIBS laser peak irradiance and related higher plasma temperature (Section 4.3) for SuperCam and ChemCam instruments,^6,23^ and a larger laser distance, leading to a larger irradiation spot of ∼2–8 mm for Raman spectroscopy, depending on measurement distance for SuperCam.^6^ The different LIBS laser parameters mean different ablation and acquisition conditions, and weak Raman signals, such as for magnetite (Figure 4e,f), may be below the detection limit. Nonetheless, obscuring of the original signals and volatile loss already occurring at low laser powers are expected to occur at higher laser powers as well, and should, therefore, be taken into account for any post-LIBS (spectroscopic) measurement.

#### 4.5.2 Space Weathering

Nanosecond pulsed lasers have been used to simulate space weathering and are able to reproduce the reddening and darkening of UV–vis–NIR reflectance spectra observed in space-weathered samples: minerals irradiated by the UV lasers at ∼ 2.0 J/cm^2^^68^ and ∼ 2.5 J/cm^2^^29^ as well as by an infrared 1064 nm laser at ∼ 20 J/cm^2^.^29^ In this study, the estimated laser fluence on the sample was ∼6 J/cm^2^, and the reported darkening^29,68^ occurs here as well. We note here that because the morphology of LIBS-formed craters, distribution of deposits, and reached temperatures on sample and ablation products depend strongly on the spatial distribution of the electric field in a laser spot,^55,69^ direct comparison between ablation experiments with different laser beam profiles, photon energies, and similar fluences is not straight forward. The postablation metrics, discussed above, may be the better criteria for such a comparison.

The main applicable set of experiments is those done under vacuum conditions because other types of weathering dominate under higher pressures, such as in Martian atmospheric conditions.^70^ In a vacuum, alteration includes a diminished signal of the original material, a polysulfide thin film, and, for pyrite only, an elemental sulfur thin film in the crater center. Here, the polysulfide thin film is likely formed through interaction with the plasma, whereas the sulfur may have been formed through fractionation within a melt layer. Considering micrometeoritic impacts as a cause of weathering, the formation of plasma is unlikely, and scattered particles likely represent the original composition of the target material;^71^ meaning that the formation of a polysulfide film is unlikely. However, it was also noted that both thin films, especially the elemental sulfur thin film, diminished significantly over a few days, a likely result of sulfur sublimation in vacuum conditions.^52^ Therefore, the additional thin film alteration in this set of experiments is not likely to be either representative of space-weathering (polysulfides) or preserved (sulfur). It should be noted, however, that other volatile-containing minerals may experience similar processes, and therefore, space-weathering may lead to volatile depletion in affected soils and meteorites.

## 5 Summary and Conclusions

This work focussed on alteration by LIBS applied to pyrite (with and without a dust layer) and pyrrhotite under different atmospheric conditions when measured through subsequent Raman spectroscopy. The alteration occurred as a side-effect of all LIBS measurements. For vacuum and Martian atmospheric conditions at 7.0 mbar, elemental sulfur was produced in pyrite, and polysulfides were produced in both pyrite and pyrrhotite. For 1.0 bar Martian atmospheric composition, magnetite was formed additionally to the sulfur and polysulfides. No additional alteration was found as a result of interaction with a ZnO dust layer.

The polysulfides and elemental sulfur indicate that laser-produced heat is the main driver for alteration under low-pressure atmospheric conditions (0–7 mbar). For higher pressures (0.007–1.0 bar), oxygen in the alteration layer is likely derived from interaction with the atmosphere. The results of this work indicate that special care must be taken with the interpretation of LIBS–Raman measurements of volatile-containing minerals, as these are thought to be highly perceptive to alteration caused by LIBS.

## Supporting Information Available

Data processing, background subtraction to account for fluorescence in Raman spectra, conversion of counts to signal-to-noise ratios, and derivation of the plasma temperature (PDF)
