# Supplementary material for: Effect of LIBS-Induced Alteration on Subsequent Raman Analysis of Iron Sulfides
Source: ACS Earth Space Chem. 2022 Aug 16;6(9):2167–79. doi: 10.1021/acsearthspacechem.2c00051 (PMC9483985; doi:10.1021/acsearthspacechem.2c00051)
Supplement: Supplementary file 1 — sp2c00051_si_001.pdf [file sp2c00051_si_001.pdf]

Supporting information

Effect of LIBS-induced alteration on subsequent Raman analysis of iron sulfides

Jitse Alsemgeest*[1]
Sergey G. Pavlov[2]
Ute Böttger[2]
Iris Weber[3]

[1]Geology and Geochemistry Cluster, Faculty of Science, Vrije Universiteit, de Boelelaan 1085, 1081HV, Amsterdam, the Netherlands
[2]Institute of Optical Sensor Systems, German Aerospace Center (DLR), Rutherfordstr. 2, 12489 Berlin, Germany
[3]Institut für Planetologie, Westfälische Wilhelms-Universität Universität Münster, Wilhelm-Klemm-Strasse 10, 48149, Münster, Germany
*E-mail: j.alsemgeest@vu.nl

Contents of this file:

Data processing: back-ground subtraction to account for fluorescence in Raman spectra, conversion of counts to signal-to-noise ratios, and derivation of plasma temperature.

Supplementary Material – Data Processing

All spectral data went through data processing before publication. In the following order, three types of data processing were performed: subtraction of dark spectra measured at the beginning of a measurement sequence, back-ground subtraction to account for fluorescence (Raman spectra only) and conversion of counts to signal-to-noise ratios (SNR). Back-ground subtraction and conversion to SNR is detailed below. For evaluation of the elemental compositions and the plasma temperature of the ablated material, original LIBS spectra were used.

Back-ground subtraction to account for fluorescence (Raman spectra only)

Back-ground subtraction was performed automatically using a script in R, written for this purpose, and the baseline.R package available through https://cran.r-project.org/web/packages/baseline/ [30-10-2021]. Baselines were calculated using an asymmetric least-square smoothing until the baseline was visibly set to a minimum, while retaining the observed peaks. In practice, this meant setting the following parameters for the baseline calculation:

1. lambda = 3
2. p = 0.05
3. maxit = 20

The entire script in R is detailed below:

```
library('baseline')
library('readxl')
library('openxlsx')

directory<-'C:/ **'%Enter directory here
normalization='off'
xlimits <- c(100,1700)
baseline_subtraction <- 'on'
filename<-'Raman Spectra All.xlsx'
inputsheet<-"Dark Subtracted (2)"
ranges=c("A6:E1029","G6:K1029","M6:Q1029","T6:X1029","Z6:AD1029","AF6:AJ1029")
%inputsheet<-"Sulfur Single Shots" %Used for calculation of sulfur spectra. Remove % at beginning of line to activate.
%ranges=c("W6:Z1029","AC6:AF1029")

numbers<-as.character(seq(1:10)-1)
myLetters<-toupper(letters[1:26])
for(i in 1:26){myLetters<-c(myLetters,toupper(paste(letters[i],letters[1:26],sep="")))}

wb = loadWorkbook(paste(directory,filename,sep='/'))
if(sum(names(wb)==paste(inputsheet,"output"))<1){addWorksheet(wb,paste(inputsheet,"output"))}
for(i in 1:length(ranges)){
  input<-read_xlsx(paste(directory,filename,sep='/'),sheet = inputsheet,range=ranges[i], col_names = FALSE)
  input_limits<-input[[1]]>xlimits[1] & input[[1]]<xlimits[2]
  input<-input[input_limits,]
  output<-input
  for(j in 2:length(input)){
    baselinetemp<-baseline(t(data.matrix(input[,j])),method='als',lambda = 3, p = 0.05, maxit = 20)
    output[,j]<-t(baselinetemp@corrected)  }
  start_range<-strsplit(ranges[i],":")
  start_range<-start_range[[1]][1]
  start_column<-start_range
```

```
  start_row<-start_range
  for(j in 1:10){    start_column<-sub(numbers[j],"",start_column) }
  start_column<-match(start_column,myLetters)
  for(j in 1:26){    start_row<-sub(myLetters[j],"",start_row)  }
  start_row<-as.numeric(start_row)
  writeData(wb,paste(inputsheet,"output"),output,startCol = start_column,startRow = start_row, colNames = FALSE)
}
saveWorkbook(wb,paste(directory,filename,sep='/'),overwrite = TRUE)
```

Conversion of counts to signal-to-noise ratios

Signal-to-noise ratios (SNR) were calculated by dividing all counts (after dark- and background subtraction) through an "average" noise value. By definition, it is impossible to determine the exact average noise as signals and noise will always be mixed. Therefore, the noise is defined in the following way (Fig. 1):

a) The difference in counts between successive points in the spectrum is calculated. This is done using: $\Delta_{Counts} = Counts_n - Counts_{n-1}$, where n represents the point in the spectrum. Logically, the average of $|\Delta_{Counts}|$ should correspond to the average noise.
b) However, as peaks (signals) are present, there is a bias towards higher $|\Delta_{Counts}|$. This becomes especially apparent when sorting $|\Delta_{Counts}|$ and plotting this.
c) To account for peaks, the highest 25% $|\Delta_{Counts}|$ are omitted. To obtain an average, the lowest 25% $|\Delta_{Counts}|$ are also omitted. The average of the middle 50% $|\Delta_{Counts}|$ (or the 2nd and 3rd quartile) gives an approximate average noise.
d) Finally, counts are divided by noise

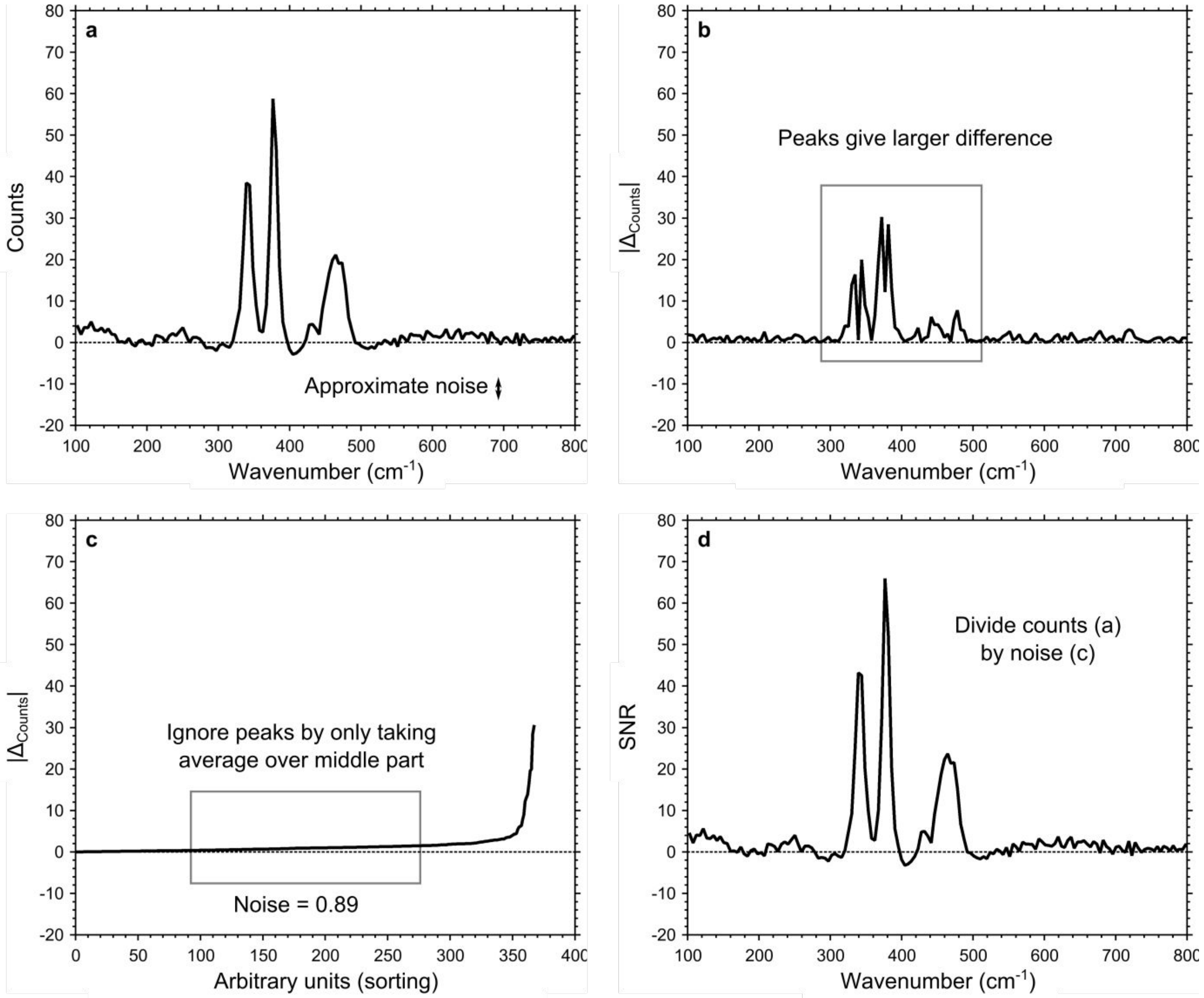

Figure S1 Conceptual model of calculation of SNR, using alteration zone C of the pyrite Raman spectrum at 7.0 mbar as example. A: Dark- and background-subtracted spectrum. B: $|\Delta_{Counts}|$ as function of wavelength. C: $|\Delta_{Counts}|$, sorted. D: Final calculated SNR.

Derivation of plasma temperature

Plasma temperatures were derived using the two-line method and the Boltzmann plots (Cremers and Radziemski, 2006). First approach is based on evaluation of relative intensities of emission lines,

namely intensity ratio of a pair of spectral lines originating in different upper levels of the same element and ionization stage and does not require knowledge on the ablation parameters. The plasma temperature was then derived using the equation:

$$T = \frac{E_i - E_m}{k \ln\left(\frac{I_{mn} g_i A_{ji}}{I_{ij} g_m A_{mn}}\right)}$$

(Miziolek et al., 2006), where the energies $E_{i,m}$ ($E_i \neq E_m$) are the upper energies; I_{mn} and I_{ij} are the integrated intensities; $g_i A_{ji}$ and $g_i A_{ji}$ are probabilities for the $n \to m$ and $j \to i$ different optical transitions, k is the Boltzmann constant.
Second approach utilizes determination of the slope of (a simple linear regression applied to) the Boltzmann plot for the linear form of the intensities I of the plasma emission lines at wavelengths λ from the excited levels with energies E_u :

$$\ln\left(\frac{I\lambda}{gA}\right) = -\frac{E_u}{kT} - \ln\left(\frac{4\pi Z}{hcN_0}\right)$$

while the intersect, depending of other parameters, such as not measured populations of the excited states, is disregarded (LIBS fundamentals and applications (Stetzler et al., 2020)).
The second approach has higher accuracy for the rich plasma spectra, extended over significant spectral range, while the first method can deliver plasma temperatures for the cases with limited spectral information, such as a few observed emission lines or if the emission transitions have very close $E_{i,m}$. / E_u.

Both approaches assume local thermal equilibrium (LTE) in the plasma plume. For the atmospheres with Martian gas compositions (at 7 mbar and 1 bar), LTE is expected to be formed after a few hundred ns (Cremers and Radziemski, 2006). It is not known whether LTE was also established at vacuum conditions. On the other hand, reduction of the emitted transitions to those with high probability, resulting in the extreme case in decrease of the spectral range towards the limited band with transitions originated from the close $E_{i,m}$. / E_u, limits the range of the upper levels so that their populations can be considered as close enough, that replaces the LTE requirements for such estimates.

Temperatures were derived mainly from the intensities of Fe neutral atomic emission lines in the spectral range 340-410 nm for the interatomic transitions (Fig. S2) with optical transition probabilities $g_i A_{ji}$ above 10^7/s, using the parameters from the NIST Atomic Spectra Database (Kramida et al., 2020)) and atomic database of the Smithsonian Astrophysical Observatory (Kurucz and Bell, 1995). For Ti, the spectral range of 300-380 nm was used, where transitions of singly ionized atoms ($g_i A_{ji}$ are above 5×10^8 /s) occur. For Zn, several lines between 330 and 640 nm with $g_i A_{ji}$ above 2×10^8 /s were observed and used for temperature estimates in the ablated ZnO powder.

The transitions pair in the two lines method were chosen to be close in emission wavelength and to be as far apart as possible in excitation energy.
It was found that the plasma temperatures derived from the Fe transitions coincide for the vacuum conditions (Table 2 in the main text). At higher pressures, Boltzmann plots can be used for accurate

estimates, while the two-line method requires manual reduction of the used line pairs by deleting a few outliers. This results in slightly (~10%) lower values of the mean temperature values. For poor spectra of Zn from the ZnO powder and Ti impurity in the sample solid matrix, the Boltzmann plots do not return reasonable values, while the two lines method offers such a possibility (see the main text for the estimates).

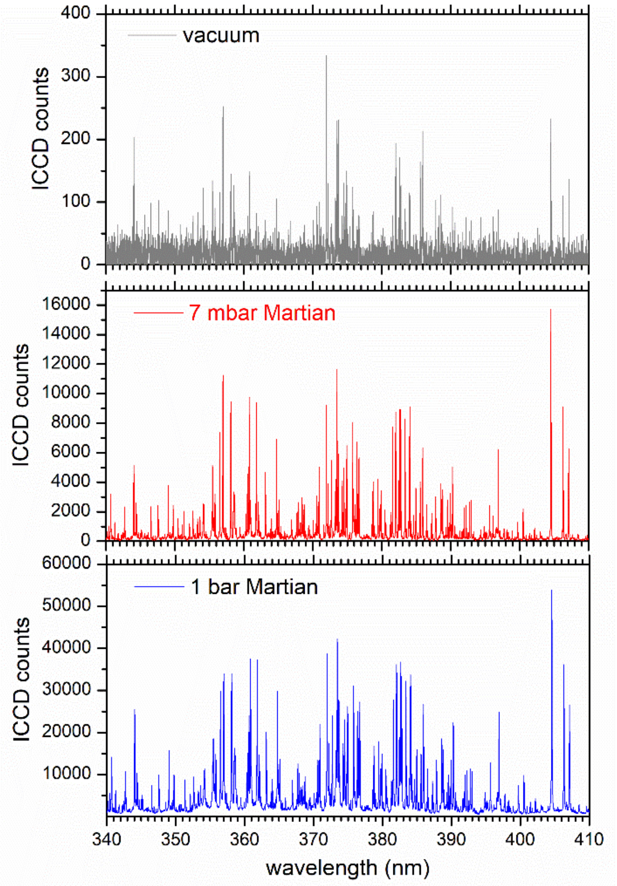

Figure S2. LIBS spectra of the pyrite sample, taken at different atmosphere and pressure, in the spectral range used for determination of the plasma temperature.

References

Cremers, D.A., Radziemski, L.J., 2006. Handbook of Laser-Induced Breakdown Spectroscopy, 1st ed. John Wiley & Sons, Ltd, Chester.

Kramida, A., Ralchenko, Y., Reader, J., NIST ASD Team, 2020. NIST Atomic Spectra Database (ver. 5.8) [WWW Document]. https://doi.org/10.18434/T4W30F

Kurucz, R.L., Bell, B., 1995. Atomic Line Data, Kurucz CD-ROM No. 23. [WWW Document]. Cambridge, Mass. Smithson. Astrophys. Obs. URL https://lweb.cfa.harvard.edu/amp/ampdata/kurucz23/sekur.html (accessed 2.11.22).

Miziolek, A.W., Palleschi, V., Schechter, I., 2006. Laser-Induced Breakdown Spectroscopy (LIBS) - Fundamentals and Applications. University Press, Cambridge.

Stetzler, J., Tang, S., Chinni, R.C., 2020. Plasma Temperature and Electron Density Spectroscopy (LIBS) in Earth ' s and Mars ' s Atmospheres. Atoms 8.
